# *siPRED*: Predicting siRNA Efficacy Using Various Characteristic Methods

**DOI:** 10.1371/journal.pone.0027602

**Published:** 2011-11-10

**Authors:** Wei-Jie Pan, Chi-Wei Chen, Yen-Wei Chu

**Affiliations:** 1 Institute of Genomics and Bioinformatics, National Chung Hsing University, Taichung, Taiwan; 2 Biotechnology Center, National Chung Hsing University, Taichung, Taiwan; 3 Institute of Molecular Biology, National Chung Hsing University, Taichung, Taiwan; 4 Graduate Institute of Biotechnology, National Chung Hsing University, Taichung, Taiwan; National Institute of Health, United States of America

## Abstract

Small interfering RNA (siRNA) has been used widely to induce gene silencing in cells. To predict the efficacy of an siRNA with respect to inhibition of its target mRNA, we developed a two layer system, *siPRED*, which is based on various characteristic methods in the first layer and fusion mechanisms in the second layer. Characteristic methods were constructed by support vector regression from three categories of characteristics, namely sequence, features, and rules. Fusion mechanisms considered combinations of characteristic methods in different categories and were implemented by support vector regression and neural networks to yield integrated methods. In *siPRED*, the prediction of siRNA efficacy through integrated methods was better than through any method that utilized only a single method. Moreover, the weighting of each characteristic method in the context of integrated methods was established by genetic algorithms so that the effect of each characteristic method could be revealed. Using a validation dataset, *siPRED* performed better than other predictive systems that used the scoring method, neural networks, or linear regression. Finally, *siPRED* can be improved to achieve a correlation coefficient of 0.777 when the threshold of the whole stacking energy is ≥−34.6 kcal/mol. siPRED is freely available on the web at http://predictor.nchu.edu.tw/siPRED.

## Introduction

It is now well established that the translation of a target mRNA can be inhibited by a small interfering RNA (siRNA). This technique, called RNA interference (RNAi), was initially described as post-transcriptional gene silencing mediated by double-stranded RNA in *Caenorhabditis elegans* [1]. RNAi can now be used to specifically suppress the expression of essentially any gene of interest. Because the siRNA is an important factor for triggering RNAi, the effectiveness of RNAi can be increased by improving the efficacy of the siRNA. In the RNAi pathway, double-stranded RNA is cleaved to yield a short, double-stranded fragment, i.e., an siRNA, by the ribonuclease III–like enzyme Dicer. Then the guide strand of the siRNA is incorporated into the RNA-induced silencing complex, which recognizes the sequence of the target mRNA by hybridization between the guide strand of siRNA and its complementary region in the target mRNA. The silencing complex then mediates the cleavage of the target mRNA to yield short fragments [2]–[4]. siRNAs of 19 nucleotides in length with 2-nucleotide 3′ overhangs are generally used for gene silencing [5].

Methods to predict the efficacy of an siRNA can be derived by analyzing various characteristics in siRNA datasets. Therefore, selecting those characteristics is crucial. We divided such predictive methods into three characteristic categories. (i) Sequence characteristics, which is based on the nucleotides as the input of the predictive system. In this case, the individual nucleotides in the sequence are transformed into numerical representations that are then used as the inputs for constructing an efficacy prediction model [6]–[8]. Moreover, the frequency of each nucleotide at a specific position is analyzed by statistical methods in a dataset, and then the conditional probability model is used to find an optimal siRNA [9]. (ii) Feature characteristics, which uses many features to predict the efficacy of an siRNA by analyzing various properties. Nucleotide composition and the stability of an siRNA or mRNA are analyzed, and then the significant features are selected to build prediction models [10]–[14]. (iii) Rule characteristics, from which the significant rules are generalized from datasets. The rules can be a basis for selecting effective siRNAs [15]–[17].

Many systems that predict siRNA efficacy have adopted a dataset comprised of mature siRNAs, i.e., the Novartis dataset, for analysis and construction. Ichihara *et al.* [18] developed the scoring method, *i-Score*, which generates a score for each nucleotide by linear regression, and then siRNA efficacy is predicted by summarizing the score. Further, the tools *Biopredsi* [19], *ThermoComposition21* [12], and *DSIR* [13] have also adopted the Novartis dataset to evolve predictive systems. *Biopredsi* applies an artificial neural network (NN) model, which yielded a high correlation coefficient of 0.66 between the observed and predicted siRNA efficacy. Because the parameters of *Biopredsi* are not demonstrated, however, Ichihara *et al.* [18] developed *s-Biopredsi*, which is similar to *Biopredsi*, and got a high correlation coefficient in predicted efficacy between *Biopredsi* and *s-Biopredsi*. The two other systems, *ThermoComposition21* and *DSIR*, use linear regression based on nucleotide preferences at each position.

In this study, we established a new system, *siPRED*, based on the Novartis dataset. *siPRED* utilizes two layers of support vector regression (SVR) to predict the efficacy of an siRNA [32]. Moreover, the Pearson correlation coefficient was applied to select the significant feature elements and the candidate characteristic methods. In the first layer, characteristic methods were derived from various characteristic categories, namely sequence, features, and rules. Further, the characteristic methods that yielded better performance were integrated into fusion mechanisms by SVR and artificial NN in the second layer. Finally, *siPRED* was used to choose the best combination of characteristic methods as the prediction model. We found that the prediction model with two layers of SVR was indeed better than each individual characteristic method, and the fusion mechanism was adopted by NN. For further analysis of the contribution of each characteristic method to the integrated method, the weight of each characteristic method in the second layer of *siPRED* was analyzed by genetic algorithms. In addition, the Matthews correlation coefficient was applied to assess the ability of *siPRED* to select highly efficacious siRNAs. The performance of *siPRED* was ≥10% higher than that of the other systems mentioned above. Finally, siRNAs could be selected based on a threshold of overall ΔG, i.e., reflecting stability of the siRNA duplex, so that the overall performance of *siPRED* for predicting siRNA efficacy was raised from 0.588 to 0.777.

## Results

### Training of characteristic methods in the first layer

In the table 1, the characteristic method F162 considered all significant feature elements in each set, whereas it did not consider the correlation between feature elements and siRNA efficacy. The other feature characteristic methods (F85, F65, F42) were constructed using different thresholds for removing various low-correlation elements.

**Table 1:** The thresholds of each feature characteristic method.

Method	S_single_	S_n-gram_	Number of features
F162	unrestricted	unrestricted	162
F85	*r* ^a^>0.10	*r* ^a^>0.09	85
F65	*r* ^a^>0.12	*r* ^a^>0.10	65
F47	*r* ^a^>0.13	*r* ^a^>0.12	47

*r* ^a^ The absolute value of correlation coefficients between feature elements and siRNA efficacy. All feature elements in S_thermodynamic_ are considered.

For investigating the accuracy of predicting siRNA efficacy for each characteristic method, the prediction models of each method were trained by SVR with 10-fold cross-validation using dataset A. The results of each characteristic method with SVR are shown in Table 2.

**Table 2:** Pearson correlation coefficient of each characteristic method trained with dataset A.

Method	*r*
Numeric	0.514
Binary	0.613
Hybrid	0.612
F162	0.602
F85	0.634
F65	0.627
F47	0.615
R12	0.569

The two sequence characteristic methods, Binary (*r* = 0.613) and Hybrid (*r* = 0.612), afforded the best training, whereas the Numeric method provided the worst training because the SVR system may have been incapable of deciphering the numerical encoding. The correlation coefficients for all feature characteristic methods were >0.6, and F85 gave the best training (*r* = 0.634), thus demonstrating that feature elements in methods could be well trained. The data presented in Table 2 show that feature characteristic methods play an important role in predicting siRNA efficacy. Moreover, the good performance of F85 indeed improved the performance of F162 because feature elements of low correlation were eliminated. The excessive reduction in feature elements in F65 and F47, however, resulted in reduced correlation coefficients, underscoring the importance of selecting a suitable number of feature elements. The rule characteristic method of training gave the worst performance because the analysis of published rules via the small dataset may have been insufficient. Consequently, the integration unit adopts only the higher correlation coefficients (*r*≥0.6) derived from the various methods, selecting the best combination of sequence and feature characteristic methods.

### Training and comparison of fusion mechanisms in the second layer

To increase the performance and accuracy of *siPRED*, the integration of sequence and feature characteristic methods established models by fusing the methods via SVR and NN. The characteristic methods having better correlation in the first layer were selected, and the integrated methods were constructed using dataset A. In addition, the prediction model was assessed with dataset B (Table 3).

**Table 3:** Pearson correlation coefficient of each integrated characteristic method.

Mechanism	Integrated method	*r* a	*r* b
SVR	Binary + F162	0.756	0.534
	Binary + F85	0.696	0.563
	Binary + F65	0.688	0.564
	Binary + F47	0.679	0.543
	Hybrid + F162	0.773	0.534
	Hybrid + F85	0.686	0.577
	Hybrid + F65	0.678	0.588
	Hybrid + F47	0.670	0.541
Neural network^c^	Binary + F162	0.783	0.566
	Binary + F85	0.691	0.579
	Binary + F65	0.686	0.585
	Binary + F47	0.680	0.580
	Hybrid + F162	0.784	0.562
	Hybrid + F85	0.685	0.579
	Hybrid + F65	0.678	0.586
	Hybrid + F47	0.670	0.580

a Pearson correlation coefficient of integrated methods trained with dataset A.

b Pearson correlation coefficient of integrated methods validated with dataset B.

c Neural network has an input layer of two nodes, a hidden layer of six nodes, and an output layer of one node.

The results of the cross-validation with dataset A showed that the correlation coefficients of integrated methods were all >0.67. The results of the fusion of methods using SVR and NN showed similar trends. However, the correlation coefficients of the integrated methods Binary+F162 and Hybrid+F162 were both >0.75, which constituted the best training with dataset A; the correlation coefficients of other integrated methods decreased gradually during training. Nevertheless, the correlation coefficients of the integrated methods compared favorably with each characteristic method, and the coefficients improved substantially during training. On the other hand, although the performance of SVR was similar to that of NN, it was apparent that the correlation coefficients with dataset A were affected by the number of feature elements, and the integrated methods with more feature elements had higher correlation coefficients. Therefore, it is clear that the feature elements play an important role in predicting siRNA efficacy.

Although the two integrated methods Binary+F162 and Hybrid+F162 gave the best training with dataset A (*r*>0.75), these two integrated methods were poorer than other integrated methods in the validation of dataset B. This revealed that overfitting occurred in the training of the integrated methods, perhaps owing to the possibility that the feature elements of F162 were relatively more compatible with dataset A. The integrated method Hybrid+F65 showed the best correlation coefficient by using SVR or NN, yielding *r* values of 0.588 and 0.586, respectively, with dataset B. Therefore, Hybrid+F65 with SVR is the major contributor to the results generated in the second layer. However, the performance was poor for the training of the single method F65 and each of F65+Hybrid and F65+Binary with dataset A, but F65+Hybrid and F65+Binary performed well in the validation with dataset B. Thus, validation with dataset B is important for selecting suitable feature elements.

Although Hybrid+F65 had a high ability to predict siRNA efficacy, the impact of each characteristic method in fusion mechanism with SVR or NN cannot be evaluated. Thus, the contribution (i.e., weight) of each characteristic method in Hybrid+F65 was evolved by genetic algorithms. The fitness function as the mean square error is: 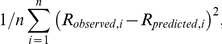



where *i* is the *i*-th siRNA in dataset A, *R_observed,i_* is the observed *i*-th siRNA efficacy, and *R_predicted,i_* is the predicted *i*-th siRNA efficacy. Further, *R_predicted_* is calculated as: 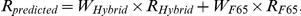



where *R_Hybrid_* and *R_F65_* are predicted outputs of the characteristic methods of Hybrid and F65, respectively, and *W_Hybrd_* and *W_F65_* are the evolved weights of Hybrid and F65, respectively. The distribution of weights in Hybrid+F65 is evident from this fitness function. Table 4 shows a steady trend of increasing *r* with the progression of generations of the training of genetic algorithms with dataset A. Furthermore, the best weights were obtained through the validation of dataset B, and a correlation coefficient of 0.57 was achieved at generation 2000. Although this coefficient was not better than that achieved with SVR or NN in training and validation, the role of Hybrid or F65 played in the integrated method could be realized. The results for *W_F65_* thus demonstrated that the feature characteristic method is significant for predicting siRNA efficacy.

**Table 4:** Determining the weights in the integrated method of Hybrid+F65 by genetic algorithms.

Generation	*W_Hybrid_*	*W_F65_*	*r*_A_	*r*_B_
100	0.179	0.814	0.670	0.569
500	0.515	0.480	0.669	0.567
1000	0.365	0.631	0.671	0.570
2000	0.359	0.637	0.671	0.570

*W_Hybrid_* belongs to the characteristic method of Hybrid, and *W_F65_* belongs to the characteristic method of F65 in the integrated method. *r*_A_ is the correlation coefficient for Hybrid+F65 trained with dataset A, and *r*_B_ was validated with dataset B. Additionally, for the genetic algorithms, the population was 100 and the rates of one-point crossover and mutation were 0.7 and 0.001, respectively.

### Comparison of algorithms

**Figure 1:**
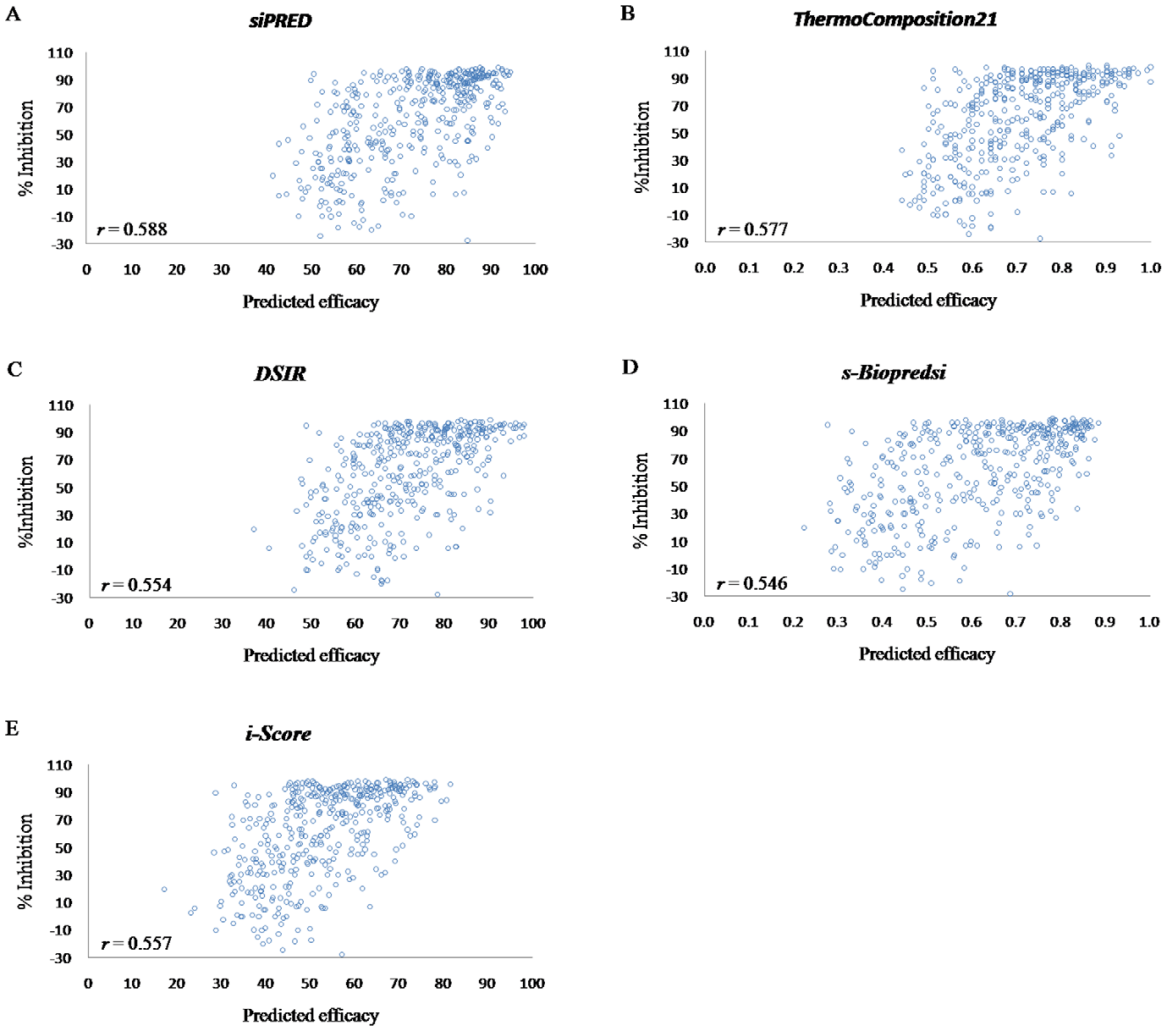
The distribution between observed and predicted siRNA efficacy using dataset B by (A) *siPRED*, (B) *ThermoComposition21*, (C) *DSIR*, (D) *s-Biopredsi*, and (E) *i-Score*. ‘*r*’ represents the Pearson correlation coefficient.

To validate *siPRED* and compare it with other systems, i.e., *i*-*Score*, *s-Biopredsi*, *ThermoComposition21*, and *DSIR*, with respect to predicting siRNA efficacy in dataset B, we obtained the results for these other systems from Ichihara *et al.* [18] (Figure 1). *siPRED* yielded a high correlation coefficient for siRNA efficacy in dataset B, which was better than that obtained with the other systems. *s-Biopredsi*, *i-Score*, and *DSIR* yielded similar correlation coefficients, namely 0.546, 0.557, and 0.554, respectively. *ThermoComposition21* and *siPRED* yielded higher (and similar) correlation coefficients of 0.577 and 0.588, respectively. *siPRED* and *ThermoComposition21* based on linear regression showed that they had better performance compared with *DSIR* as well as *s-Biopredsi* using NN and *i-Score* using the scoring method.

In addition, Accuracy (Acc), sensitivity (Sn), specificity (Sp), and the Matthews correlation coefficient (MCC) were used to evaluate the predictive ability of each system. Four measures were defined: 
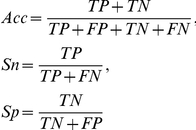



and 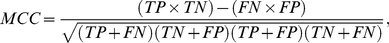



where TP, FP, FN and TN are true positives, false positives, false negatives, and true negatives, respectively. Sn and Sp represent the rate of true positives and true negatives respectively. Acc is the overall accuracy of prediction. Additionally, MCC is a measure of the quality of the classifications, and the value may range between −1 (an inverse prediction) and +1 (a perfect prediction), with 0 denoting a random prediction.

In dataset B, siRNAs of high efficacy (≥70% inhibition of target mRNA) were selected to analyze each system. The trend in MCC was as follows: *siPRED*>*DSIR*>*ThermoComposition21*>*s-Biopredsi*>*i-Score* (Table 5). *siPRED* performed well, with MCC = 0.517 and Acc = 75.7%, and MCC improved at least 10% more than for the other predictive systems. The Sn of *i-Score* was 15.5%, indicating a low rate of false negative siRNAs, and the best Sn value of all the other systems (74.2%) was lower than that of *siPRED* (83.1%). Therefore, *siPRED* showed the highest rate of predicting high-efficacy siRNAs. Additionally, *i-Score* had a high specificity of 97.1%, and thus it is possible that *i-Score*, in its current form, may actually be helpful for rejecting low-efficacy siRNAs. Furthermore, *siPRED* had relatively low specificity (67.96%), indicating that this aspect of *siPRED* must be improved. On the other hand, the correlation coefficient is a measure of the accuracy of predicting siRNA efficacy, and *siPRED* had a slightly higher correlation coefficient than the other systems (Figure 1). However, both Acc and MCC can distinguish between high- and low-efficacy siRNAs.

**Table 5:** Performance of each system for predicting siRNA efficacy (i.e., ≥70% inhibition of the target mRNA).

Predictive system	Acc (%)	Sn (%)	Sp (%)	MCC
*siPRED*	75.66	83.10	67.96	0.517
*i-Score*	55.61	15.49	97.09	0.216
*s-Biopredsi*	67.30	55.40	79.61	0.360
*ThermoComposition21*	70.41	72.77	67.96	0.407
*DSIR*	70.64	74.18	66.99	0.412

We next determined the correlation (*r* = −0.28) between base stacking energy for the 19-nucleotide siRNA sequence (the whole ΔG) and the efficacy with which an siRNA could inhibit its target mRNA. Ichihara *et al.* [18] calculated a threshold of −34.6 kcal/mol for the whole ΔG [18] and used this threshold to divide dataset B into two sets. Their predictive system, the utilization of the two sets (i.e., ΔG<−34.6 kcal/mol or ≥−34.6 kcal/mol) actually improved the correlation coefficient. Thus, *siPRED*, *i-Score*, *s-Biopredsi*, *ThermoComposition21*, and *DSIR* were applied to the two sets with the threshold of the whole ΔG, and the results are shown in Table 6. The set comprising siRNAs with ΔG≥−34.6 kcal/mol totaled 101 siRNAs. Using this set, *siPRED* yielded the highest correlation coefficient (*r* = 0.777), and the *r* for *i-Score* actually improved from 0.557 to 0.723; this was also the case for the other systems, especially *s-Biopredsi* and *DSIR*, for which *r* increased from 0.546 to 0.724 and from 0.554 to 0.733, respectively. The set comprising siRNAs with ΔG<−34.6 kcal/mol totaled 318 siRNAs; when this set was used, *s-Biopredsi* and *DSIR* had an *r* of <0.5, whereas *siPRED*, *i-Score*, and *ThermoComposition21* had an *r* of >0.5. Furthermore, the *r* of *siPRED* (0.538) was slightly less than that of *ThermoComposition21* (0.551). Thus, the accuracy of *siPRED* could be further enhanced by improving the analysis of the whole ΔG.

**Table 6:** Effect of the whole ΔG for each system on prediction accuracy.

Predictive system	*r* a	*r* b
*siPRED*	0.777	0.538
*i-Score*	0.723	0.514
*s-Biopredsi*	0.724	0.498
*ThermoComposition21*	0.677	0.551
*DSIR*	0.733	0.499

a Pearson correlation coefficient of siRNAs (total of 101) having a ΔG threshold ≥−34.6 kcal/mol with dataset B.

b Pearson correlation coefficient of siRNAs (total of 318) having a ΔG threshold <−34.6 kcal/mol with dataset B.

## Discussion

A high correlation coefficient for predicting siRNA efficacy can be achieved by combining characteristic methods with fusion mechanisms. In the first layer of *siPRED*, however, it is very important to select appropriate feature elements. The feature characteristic method F162, in which feature elements were significant (p-value<0.001) but did not further select elements of high correlation, had the worst performance (Table 2). Therefore, we assumed that the feature elements of higher correlation could increase the training ability of each feature characteristic method. Although this was indeed the case, especially for F85 for which the correlation coefficient increased by 5.3%, selecting feature elements of higher correlation for F47 increased the correlation coefficient by only 2.1%. Because F47 and F162 gave similar results, our assumption is imperfect. If feature elements are not correctly selected, it is possible that there is a fault in the system that lowers the correlation coefficient of the predictive system. Consequently, the best strategy is to eliminate feature elements of low correlation by conducting an analysis based on a correlation threshold in the first layer.

Based on the values of the various correlation coefficients of all the characteristic methods in the second layer (Table S1), high correlations were found among each method for predicting siRNA efficacy, with the exception of F162. The highest correlation coefficient for F162 was achieved between the predicted and observed efficacy (*r* = 0.782). This could cause the integrated methods, including F162, to yield a high correlation coefficient during training with dataset A, although the worst performance in validation was with dataset B. This phenomenon is caused by overfitting because F162 has too many feature elements. Furthermore, F162 has a higher correlation coefficient with F85 compared with the other characteristic methods. This observation reveals that the predicted efficacy of F85 is similar to that of F162 and that F85 is also likely to incur a certain degree of overfitting. For this reason, integrated methods, including F85, did not perform best with dataset B in fusion mechanisms. Thus, the best method for use in *siPRED* is Hybrid+F65.

Additionally, thermodynamic parameters were notably important in the study of Ichihara *et al.* [18] and Lu *et al.* [14]; in particular, Lu *et al.* considered many kinds of thermodynamic parameters to select effective siRNAs. Therefore, we also adopted thermodynamic parameters even through *n*-grams had provided above 50% feature elements in feature characteristic methods. Even so, Sp in our study did not have the best performance compared with other predictive systems, and *siPRED* might be improved by considering more thermodynamic parameters.

We have created a freely available web tool for *siPRED* (http://predictor.nchu.edu.tw/siPRED). The input of the *siPRED* server is the sequence of the target mRNA. The nucleotides of A, U/T, C and G are acceptable in the input sequence, and the threshold setting of siRNA efficacy is also provided. Upon completion of the predictive processing, the results are presented in three parts. One part shows all candidate siRNAs above the threshold, and the other parts are used with the whole ΔG to divide the candidate siRNAs into two sets. The first set contains candidate siRNAs of high predictive accuracy as determined by *siPRED*, and the second set contains a little siRNAs of high efficacy, but the predictive accuracy in the second set is lower than that in the first set. We suggest selecting the first set, which contains candidate siRNAs having a ΔG≥−34.6 kcal/mol.

## Materials and Methods

### Datasets

Two siRNA datasets were used to construct and validate *siPRED* for predicting siRNA efficacy. Dataset A, consisting of 2431 siRNAs that Husken *et al.* [19] verified by experiment, was the foundation for establishing *siPRED*. Dataset B, a testing dataset, was mutually exclusive with dataset A, and it was used to validate the predictive systems. Dataset B consisted of 419 siRNAs that Ichihara *et al.* [18] gathered from five reports that each reported a small num­ber of siRNAs [5], [17], [20]–[22]. Because dataset B differed from dataset A, it was used to estimate the accuracy of the prediction systems.

### Three categories of characteristic methods

#### Sequence characteristics

The first category includes numeric, binary, and hybrid encoding characteristic methods. Numeric encoding is applied to the nucleotides A, U, C and G, each of which is assigned a number 1, 2, 3 and 4, respectively. Binary encoding is A = 0 0 0 1, U = 0 0 1 0, C = 0 1 0 0 and G = 1 0 0 0. To increase the specificity of encoding, hybrid encoding is used with binary encoding and a weight, which is a correlation coefficient between the nucleotide at each position and the observed siRNA efficacy (Figure S1). For example, the hybrid encoding for the adenine nucleotide A*_n_* is 0 0 0 1 *p_n_*, where *p_n_* is the correlation coefficient between adenine at the *n*-th position and the siRNA efficacy.

#### Feature characteristics

The second category of characteristic methods was developed by analyzing and selecting the significant features (p-value <0.001) as elements in three kinds of sets, namely a single nucleotide, nucleotide composition, and thermodynamic parameters. Each characteristic method adopts a different number of feature elements, which could be utilized as the input of SVR. The first set, S_single_, comprises the feature elements as the nucleotide at a specific position. If the nucleotides of the input sequence are fitted with feature elements in S_single_, the corresponding input of SVR has a value of 1. By contrast, when the nucleotide of the input sequence is not matched, the value is 0. For the nucleotide composition set, the *n*-gram approach [23], [24] was adopted to fetch each possible subword, which is a given nucleotide string with length *n* (*n* = 2, 3, 4 or 5) from the siRNA sequence. Most of the previous studies have considered only the presence or absence of a subword. However, we added the concept of frequency to the *n*-gram approach. Therefore, a correlation coefficient was obtained according to the frequency of a certain nucleotide composition within a given sequence and siRNA efficacy. Only the significant feature elements of nucleotide composition were selected as the second set S*_n_*_-gram_ to increase the confidence and discrimination. For example, if S*_n_*_-gram_ includes the feature element AG, and if the sequence is AGGAG, the frequency of AG is 2.

siRNA stability greatly influences the prediction of siRNA efficacy [21], [25]. Therefore, the feature elements in the third set are acquired from thermodynamic parameters, considering the stacking energies spanning the antisense positions 1–2, 2–3, …, 18–19 as reflecting the internal stability, the sum of stability at positions 1–19, ΔH, and ΔS by using a nearest neighbor model [26]. Because most feature elements in the third set are statistically significant of correlations (*r*>0.1), all S_thermodynamic_ values would be used to design feature characteristic methods.

The three feature sets were established after analyzing single nucleotides, nucleotide composition, and thermodynamic parameters, and then the different sets were combined to develop four characteristic methods (Table 1).

#### Rule characteristics

Such rules for the mapping of nucleotide preference and siRNA efficacy were formulated based on published reports [5], [12], [16], [17], [27]–[31] (Table S2) and were validated by an analysis based on information known about siRNAs. The mapping table includes 12 rule sets and assists with the input of data for the characteristic method, in which the nucleotide at each position is mapped into 12 inputs with rule sets. The encoding is 1 if the nucleotide is mapped with high efficacy based on the rules. By contrast, the encoding is −1 if the efficacy is low, and encoding is 0 if no rules are followed. For example, if there is an adenine at the third position, which satisfies the high-efficacy rule in the rule set of Reynolds, the corresponding encoding will be set to 1. If the low-efficacy rule in the rule set of Amarzguioui is also satisfied, the corresponding encoding will be set to −1. If the remainders of the rule sets are not satisfied, the inputs of adenine will get ten 0 s.

### Construction of siPRED

*siPRED* comprises two layers that are designed to yield a high correlation coefficient between observed and predicted siRNA efficacy. The predicted ability of each single characteristic method is realized after the first layer by training in various categories. Because the integration selected that the characteristic methods with better correlation coefficient are more influential, the overall performance is more possible to improve. The characteristic methods with better correlation coefficients are selected and integrated into the second layer for improving overall performance. Moreover, the fusion mechanisms with SVR and NN in the second layer are used to compare with each other by performance improvement. However, the algorithms are a “black box”, and the effect from the characteristic methods is not observed in the fusion mechanism. Thus, the integrated methods having the best performance are selected to analyze the degree of influence in the fusion mechanism by genetic algorithms.

## Supporting Information

Figure S1 **Pearson correlation coefficient between nucleotide composition of position specific and observed siRNA efficacy.**
(TIF)

Table S1 **Correlation coefficients among characteristic methods and correlation coefficients between each characteristic method and actual siRNA efficacy.** The correlation coefficients were computed using dataset A.
(PDF)

Table S2 **The preference for nucleotides at each position in the sense strand as taken from published reports.**
(PDF)
