# Supplementary figures and images for: siPRED: Predicting siRNA Efficacy Using Various Characteristic Methods
Source: PLoS One. 2011 Nov 10;6(11):e27602. doi: 10.1371/journal.pone.0027602 (PMC3213166; doi:10.1371/journal.pone.0027602)

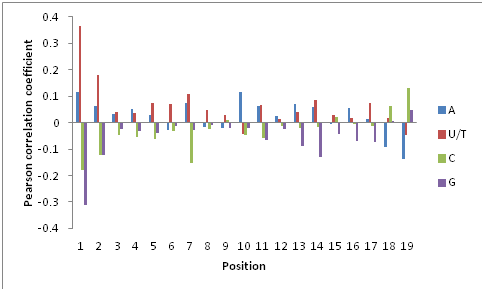

Supplement: Figure S1 — Pearson correlation coefficient between nucleotide composition of position specific and observed siRNA efficacy. (TIF) [file pone.0027602.s001.tif]
