# Supplementary material for: siPRED: Predicting siRNA Efficacy Using Various Characteristic Methods
Source: PLoS One. 2011 Nov 10;6(11):e27602. doi: 10.1371/journal.pone.0027602 (PMC3213166; doi:10.1371/journal.pone.0027602)
Supplement: Table S1 — Correlation coefficients among characteristic methods and correlation coefficients between each characteristic method and actual siRNA efficacy. The correlation coefficients were computed using dataset A. (PDF) [file pone.0027602.s002.pdf]

Table S1. Correlation coefficients among characteristic methods and correlation coefficients between each characteristic method and actual siRNA efficacy.

	Binary	Hybrid	F162	F85	F65	F47
Binary	1					
Hybrid	0.997	1				
F162	0.858	0.847	1			
F85	0.922	0.921	0.902	1		
F65	0.920	0.919	0.887	0.989	1	
F47	0.926	0.925	0.876	0.980	0.990	1
Actual efficacy	0.668	0.646	0.782	0.679	0.665	0.656

The correlation coefficients were computed using dataset A.
