# Supplementary material for: siPRED: Predicting siRNA Efficacy Using Various Characteristic Methods
Source: PLoS One. 2011 Nov 10;6(11):e27602. doi: 10.1371/journal.pone.0027602 (PMC3213166; doi:10.1371/journal.pone.0027602)
Supplement: Table S2 — The preference for nucleotides at each position in the sense strand as taken from published reports. (PDF) [file pone.0027602.s003.pdf]

Table S1. The preference for nucleotides at each position in the sense strand as taken from published reports.

Position / Rules	1				2				3				4				5				6			
	A	C	G	U	A	C	G	U	A	C	G	U	A	C	G	U	A	C	G	U	A	C	G	U
**Reynolds**									●															
**Ui-Tei**	▼	●	●	▼																				
**Amarzguioui**	▼	●	●	▼	▼			▼	▼			▼									●			
**Hsieh**																						▼		
**Takasaki**	▼		●	▼																	●	▼		
**Svetlana**	▼		●	▼			●				●	▼		▼							●	▼	▼	●
**Jagla 1**		●	●																					
**Jagla 2**		●	●																					
**Jagla 3**		●	●																					
**Jagla 4**	●			●													●			●	●			●
**Matveeva**	▼	●	●	▼				▼		▼						●					●	▼		●
**Jiang**			●	▼			●				●	▼									●	▼	▼	●

● Preference for high siRNA efficacy. ▼Preference for low siRNA efficacy.

Position / Rules	7				8				9				10				11				12			
	A	C	G	U	A	C	G	U	A	C	G	U	A	C	G	U	A	C	G	U	A	C	G	U
**Reynolds**																●								
**Ui-Tei**																								
**Amarzguioui**																								
**Hsieh**																		●	●					
**Takasaki**			●	▼	●		▼				▼	●												
**Svetlana**	●	▼										▼				●								
**Jagla 1**													●			●								
**Jagla 2**														●	●									
**Jagla 3**																		●	●					
**Jagla 4**																								
**Matveeva**	●	▼									▼					●					●		▼	
**Jiang**	●	▼										▼				●								

Position / Rules	13				14				15				16				17				18			
	A	C	G	U	A	C	G	U	A	C	G	U	A	C	G	U	A	C	G	U	A	C	G	U
**Reynolds**			▼																					
**Ui-Tei**																								
**Amarzguioui**																	●			●	●			●
**Hsieh**															●									
**Takasaki**												●												
**Svetlana**	●	▼	▼	●		▼			●			▼					●				●	▼		
**Jagla 1**																								
**Jagla 2**																								
**Jagla 3**																								
**Jagla 4**																								
**Matveeva**	●			●						▼						●	●		▼		●	▼	▼	
**Jiang**	●	▼	▼	●		▼			●			▼					●				●	▼		

Rules \ Position	19			
	A	C	G	U
**Reynolds**		▼	▼	
**Ui-Tei**	●	▼	▼	●
**Amarzguioui**	●		▼	●
**Hsieh**			▼	●
**Takasaki**			▼	
**Svetlana**	●		▼	●
**Jagla 1**	●			●
**Jagla 2**	●			●
**Jagla 3**		●	●	
**Jagla 4**	●			●
**Matveeva**	●	▼	▼	●
**Jiang**	●	▼	▼	●
